# How circadian variability of the heart rate and plasma electrolytes concentration influence the cardiac electrophysiology – model-based case study

**DOI:** 10.1007/s10928-021-09744-1

**Published:** 2021-03-05

**Authors:** Barbara Wiśniowska, Zofia M. Bielecka, Sebastian Polak

**Affiliations:** 1 Faculty of Pharmacy, Jagiellonian University Medical College, Medyczna 9 Street, 30-688, Kraków, Poland; 2 Simcyp Division, Certara UK Limited, Level 2-Acero, 1 Concourse Way, Sheffield, S1 2BJ UK

**Keywords:** Cardiac safety, Cardiac Safety Simulator, Circadian rhythm, Intrasubject variability, QTc

## Abstract

The circadian rhythm of cardiac electrophysiology is dependent on many physiological and biochemical factors. Provided, that models describing the circadian patterns of cardiac activity and/or electrophysiology which have been verified to the acceptable level, modeling and simulation can give answers to many of heart chronotherapy questions. The aim of the study was to assess the performance of the circadian models implemented in Cardiac Safety Simulator v 2.2 (Certara, Sheffield, UK) (CSS), as well as investigate the influence ofcircadian rhythms on the simulation results in terms of cardiac safety. The simulations which were run in CSS accounted for inter-individual and intra-individual variability. Firstly, the diurnal variations in QT interval length in a healthy population were simulated accounting for heart rate (HR) circadian changes alone, or with concomitant diurnal variations of plasma ion concentrations. Next, tolterodine was chosen as an exemplary drug for PKPD modelling exercise to assess the role of circadian rhythmicity in the prediction of drug effects on QT interval. The results of the simulations were in line with clinical observations, what can serve as a verification of the circadian models implemented in CSS. Moreover, the results have suggested that the circadian variability of the electrolytes balance is the main factor influencing QT circadian pattern. The fluctuation of ion concentration increases the intra-subject variability of predicted drug-triggered QT corrected for HR (QTc) prolongation effect and, in case of modest drug effect on QTc interval length, allows to capture this effect.

## Introduction

It is a well-known fact that dynamic physiology modifications in all living systems are not only responses to the changing environment but also are the effects of circadian rhythms which are expressions of a so-called “physiological clock” [1, 2] These molecular level endogenous changes can be observed as daily fluctuations of such clinical parameters as body temperature, blood pressure, urine excretion, or cardiac electrophysiology. Circadian rhythm of the latter, namely the cardiac electric activity, is physiologically and biochemically based; it is attributed to diurnal fluctuations at the level of the balance between two limbs of the autonomic nervous system, catecholamine levels, blood pressure, plasma ion concentrations, and ion channels expression and activity. There are well-established models describing cardiac electric activity, whose parameters may be influenced by xenobiotics and at the same time are time-dependent. Provided, that there are models describing the circadian patterns of cardiac activity and/or electrophysiology which have been verified to the acceptable level, modeling and simulation can give answers to many heart chronotherapy questions. The aim of the study was: (1) the evaluation of the performance of the circadian models implemented in the Cardiac Safety Simulator v 2.2 (Certara, Sheffield, UK) (CSS) – a modeling and simulation-based platform for the assessment of pro-arrhythmic potency of xenobiotics, and (2) the assessment of how the accounting for circadian rhythms in modelling and simulation would affect the simulation results in terms of cardiac safety.

As it was stated before, the model parameters that refer to physiological variables contributing to the normal cardiac electrophysiology, are time-dependent. The circadian patterns of the changes of the values of these variables have been investigated in many studies. For example, it was observed that the lowest potassium level is achieved at 9 pm and then rises slowly to reach the highest level at around 1 pm [3]. Also, ionic channel gene expression is not constant in time. 24-h pattern in the expression of genes of potassium and sodium cardiac channels contributing to cardiomyocyte molecular clock was observed [4–6]. As for physiological regulation, the circadian rhythm of autonomic nervous system activity drives the circadian rhythm of the heart rate (HR), which parallels with the 24-h cycle of blood pressure [7]. Also, ECG indices such as P wave duration and its area, PR interval, QRS complex, and QT interval follow circadian patterns and depend on heart rate rhythmicity. Thus, the values of these ECG measurements mirror the autonomic nervous system activity; they decrease in the daytime and increase in the night time, when the sympathetic or parasympathetic system dominates, respectively. These circadian rhythms are mirrored not only in the patterns of normal cardiac electrophysiology, but also in pathophysiological heart activity as well. Since some of the conditions exhibiting circadian patterning are more predisposing to arrhythmias than others, the circadian variation in arrhythmias and sudden cardiac death occurrence can be observed. However, there are many other arrhythmogenic factors independent of any physiological clock that confound the observations. Portaluppi et al. reviewed circadian patterns of different cardiac arrhythmias in [7]. Prolongation of myocardial repolarization manifested as QT interval prolongation in the ECG is one of the three most common types of repolarization modifications predisposing to ventricular arrhythmias [8]. QT interval and QT dispersion showed a day-night pattern in many studies [9–12]. Longer QT interval were observed during sleep than during the daytime, but the QT dispersion was significantly greater during the day. However, because QT interval is inversely related to the heart rate, in clinical practice the heart-rate corrected QT (or QTc), is often used and is more clinically meaningful. The relationship between the heart rate and QT interval length is complex; therefore a number of heart rate correction formulae have been proposed and used in clinical practice [13]. Consequently, the extent of circadian changes in QTc depend on the used formula leading to over- or underestimation of daily variation of this parameter values as well as the drug-induced QTc prolongation [11, 12, 14].

From the practical point of view, knowledge of the circadian patterns of cardiac rhythm opens the door to chronotherapy of the rhythm disruptions. This can be done by application of a drug in a specific dosing regimen [7] or pharmacological modulation of the circadian clock [8] or prediction of drug cardiac effect in terms of its safety. It may be preceded by modeling and simulation, which can be a useful approach in chronotherapy, provided that the tools used for this purpose offer verified circadian models.

## Methods

The mathematical models for circadian rhythms of heart rate and cations concentrations in healthy individuals used in this study have been published previously [15]. In brief, the PhysioBank data warehouse and the Cracow’s clinical research database (1st Department of Cardiology and Hypertension, Jagiellonian University Medical College) were used to retrieve a set of experimental observations for the circadian changes of the heart rate and to create and verify a multivariate linear regression model of the relationship between RR interval (dependent variable) and a set of independent variables i.e. age, sex and the time of measurement. The final model was formulated as follows (Eq. 1):

1 $$\log \left( {RR} \right) = 7.163 + 0.0961 \times Sex - 0.0243 \times Age\; 0.00027 \times Age^{2} + 0.1055 \times \sin \left( {\frac{2\pi }{{24}} \times Hour} \right) + 0.0664 \times cos\left( {\frac{2\pi }{{24}} \times Hour} \right) - 0.0155 \times \sin \left( {\frac{2\pi }{{24}} \times Hour} \right) \times Sex + 0.0608 \times \cos \left( {\frac{2\pi }{{24}} \times Hour} \right) \times Sex$$

where Sex – 1 for male, 0 for female, Age – age in years, Hour – a time of a day (0–24).

To develop models of the circadian rhythm of potassium, sodium, and calcium concentrations in plasma, the models proposed by Sennels et al. [16] were extensively modified and verified based on data for a large number of healthy subjects of both sexes and a wide range of ages [17, 18]. The mean ion concentrations at the time of a day were calculated by the following formulas (Eq. 2):

2 $$\begin{gathered} mean\left[ {K^{ + } } \right] = mean_{F/M} \left[ {K^{ + } } \right] + 0.18 \times \cos \left( {\frac{2\pi }{{24}} \times Hour - 10:07} \right) \hfill \\ mean\left[ {Na^{ + } } \right] = mean_{F/M} \left[ {K^{ + } } \right] + 1.1 \times \cos \left( {\frac{2\pi }{{24}} \times Hour - 13:08} \right) \hfill \\ mean\; logit{ - }transformed\left[ {Ca^{2 + } } \right] = mean_{F/M}\; logit{ - }transformed\left[ {Ca^{2 + } } \right] \hfill \\ \end{gathered}$$

where M/F – male or female, Hour – a time of a day (0–24).

Mean ion concentrations:

K^+^ [mM]: female 4.088; male 4.213Na^+^ [mM]: female 138.169; male 140.096.

Ca^2+^ [mM]: female 2.313, male 2.418 (logit-transformed: female −0.5, male 0.1

All the developed models are implemented into Cardiac Safety Simulator, a platform for cardiac electrophysiology simulations used in the current study. CSS combines biophysically detailed models of cardiac cells with the database of human physiological, genotypic, and demographical data; thus, it allows to introduce interindividual variability into the simulations and assess its influence on the ECG parameters [19]. Incorporation of models of circadian rhythmicity of HR and ion concentrations introduces also intrasubject variability to the simulations and makes them closer to the real, clinical situation.

The population under the study is composed by the virtual population generator [20] based on the demographic information (gender, age, and body weight) and bootstrapping method. The model assigns cardiomyocyte volume, area, electrical capacitance based on the age of the included subjects.

In the current study, the cardiomyocyte electrophysiology and its drug-triggered modifications were simulated using the well-established ten Tusscher 2006 human action potential model [21] implemented in the CSS platform. The ten Tusscher model reproduces a physiological process of action potential generation based on the description of major cardiac ion currents, i.e.: fast sodium, L-type calcium, transient outward, rapid and slow delayed rectifier, and inward rectifier potassium currents and a basic calcium dynamics. To reflect the non-homogenous composition of the ventricular wall, the models of endocardial, mid-myocardial, and epicardial cells (50:30:20) were connected into a one-dimensional string. The fibre, with the age and gender-specific length, is paced at the epicardial side with an average diffusion coefficient of 0.0016 cm^2^/ms. A space step and a time step were set to ∆x = 0.01 mm and ∆t = 0.01 ms, respectively, and total simulation time was set to 10,000 ms.

The clinical ECG data described by Smetana et al. [12] in the study involving a population of 53 healthy volunteers were used for the verification of a performance of the model of circadian HR fluctuations. The observed values were digitized manually from the plot with the use of GetData Graph Digitizer. The virtual study parameters were set to mimic clinical study i.e. number of subjects equalled 53 and the proportion of females was 0.53. The age of subjects ranged from 18 to 49 years. PseudoECG signals were simulated for 24 h period and uncorrected QT values were compared with those registered during clinical observation. Furthermore, an individual QT correction (for Smetana study) or study specific correction (Malhotra study) was used by fitting the parameter ‘n’ in the following formula to the data generated by simulating the QT and RR (Eq. 3):

3 $$QTcI\; or\; QTcS = \frac{QT}{{RR^{n} }}$$

Tolterodine was chosen as an exemplary drug to assess the role of circadian rhythmicity in the electrophysiological simulations and to investigate the drug effects on a QT interval. For comparison and evaluation of the virtual study results, relevant data from a thorough QT study determining the QTc effects of two dose levels (recommended - 2 mg BID and supra-therapeutic - 4 mg BID) of tolterodine, reported by Malhotra et al. [22] was extracted. The design of the virtual study was mimicked in terms of population size, age, and gender distribution, dose, and QT interval measurement time points according to the real clinical settings. Individual, time-specific tolterodine unbound plasma concentrations were simulated with the use of Simcyp simulator version 19 (Certara, Sheffield, UK) and built-in tolterodine pharmacokinetic model. The details of the PK modelling of tolterodine can be found elsewhere [23].

Simulated plasma concentrations were transferred as the inputs to CSS and translated into corresponding current inhibition. The current inhibition extent was calculated based on in vitro experimental data collected from available literature sources (Table 1) [24, 25] with the use of the Hill equation. Based on drug-dependent currents changes the pseudoECG signal was simulated with the use of CSS.Based on obtained data Fridericia’s and study-specific heart rate-corrected QT values (QTcF and QTcS) were calculated and placebo and drug-related changes (ΔQTcF/S) were established.

**Table 1:** Tolterodine inhibitory potential against four main cardiac ion channels: IKr (encoded by hERG gene), delayed rectifier potassium current IKs (encoded by KvLQT1/mink gene), peak sodium current INa (encoded by Nav1.5 gene), L-type calcium current ICa (encoded by Cav1.2 gene)

	IC_50_ [µM]	Hill coefficient	In vitro model	Temperature	Source
IKr	0.0096	1.09	HEK	physiological	[31]
IKs	79.43	1	CHO	room	[32]
INa	19.12^a^	1	CHO	room	[32]
ICa	25.12	1	HEK	room	[32]

The potency of a substance was assessed as the half-maximal inhibitory concentration (IC_50_) in either Human embryonic kidney 293 (HEK) or Chinese hamster ovary (CHO) cell model

^a^Mean value from 2 in vitro studies

## Results and discussion

**Fig. 1:**
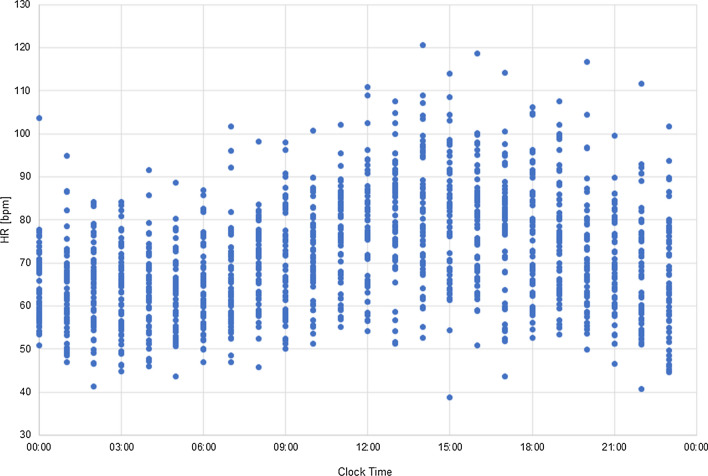
Simulated diurnal variation of the heart rate, presented as RR interval length [s] at all hours of day and night, in the virtual population

**Fig. 2:**
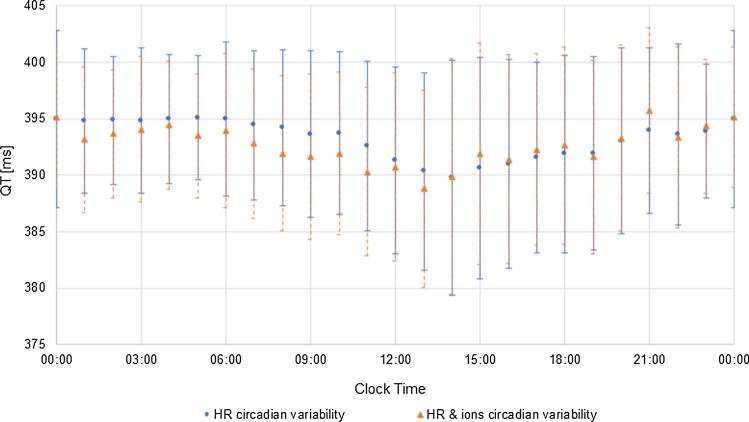
Simulated diurnal variation of QT interval length values [ms] in the virtual population. Data are presented as the mean values with a standard deviation of the mean. The blue dots represent the mean QT values when the HR circadian changes alone were accounted for in the simulation scenario. Orange triangles represent the mean QT values when both, the HR circadian changes and plasma ion concentrations changes were accounted for in the simulation scenario

The generated virtual population consisted of 53 virtual subjects (28 women, age 28.5  ±  7.3 and 25 men, age 30.3  ±  8.8). The pseudoECG signals were generated every hour from 00:00 to 24:00 (24 time points). Figures 1 and 2 present the hourly simulated diurnal variations in HR and QT interval values accounting for HR circadian changes alone or with concomitant plasma ion concentrations diurnal variations, respectively.

The RR and QT intervals are sinusoidal in character with a period of around 24 h. The changes in the mean QT intervals reflected the circadian variations of the RR interval and both intervals were longer during usual sleep time hours and shorter during daytime activity hours. It is in line with the observations that the sleep time, which is associated with sympathetic withdrawal, is related to slowing of the heart rate [26] and prolongation of QT interval [10]. In general, QT alterations were similar when accounting for HR changes only or both HR and ion fluctuations, however, the introducing different values of K^+^, Na^+^, and Ca^2+^ concentration increased intra-individual diurnal QT variability (average SD = 6.1 ms vs 3.3 ms for HR and HR plus ion circadian changes, respectively).

The 24-h variability of the simulated QT intervals followed the daily variations observed by Smetana et al. [12]. For individual subjects, the diurnal variation of QT values ranged from ~ 10 to 47 ms, with a mean daily amplitude of 24 ms. The mean QT difference in daytime and night-time as reported by Smetana et al. was 29.7 ms. However, the mean simulated QT values at defined time points and their daily amplitude were much smaller due to large interindividual simulated QT interval variability in the virtual population for each timepoint.

The QT interval length is closely related to the heart rhythm. Therefore, to investigate real daytime changes, the QT interval needs to be corrected for the HR allowing for observation of the QT circadian rhythmicity independently of the heart rate. All prospective correction formulas are built under the assumption that there is a QT-RR relation that can be described by the mathematical expression universal for all individuals. However, it is well known that different correction formulas give inconsistent results. It suggests that although the relationship between QT and RR exists, it is highly individual. Thus for the detailed assessment of QTc interval length and its changes due to different risk factors an application of the subject-specific heart rate correction is needed [27, 28].

**Fig. 3:**
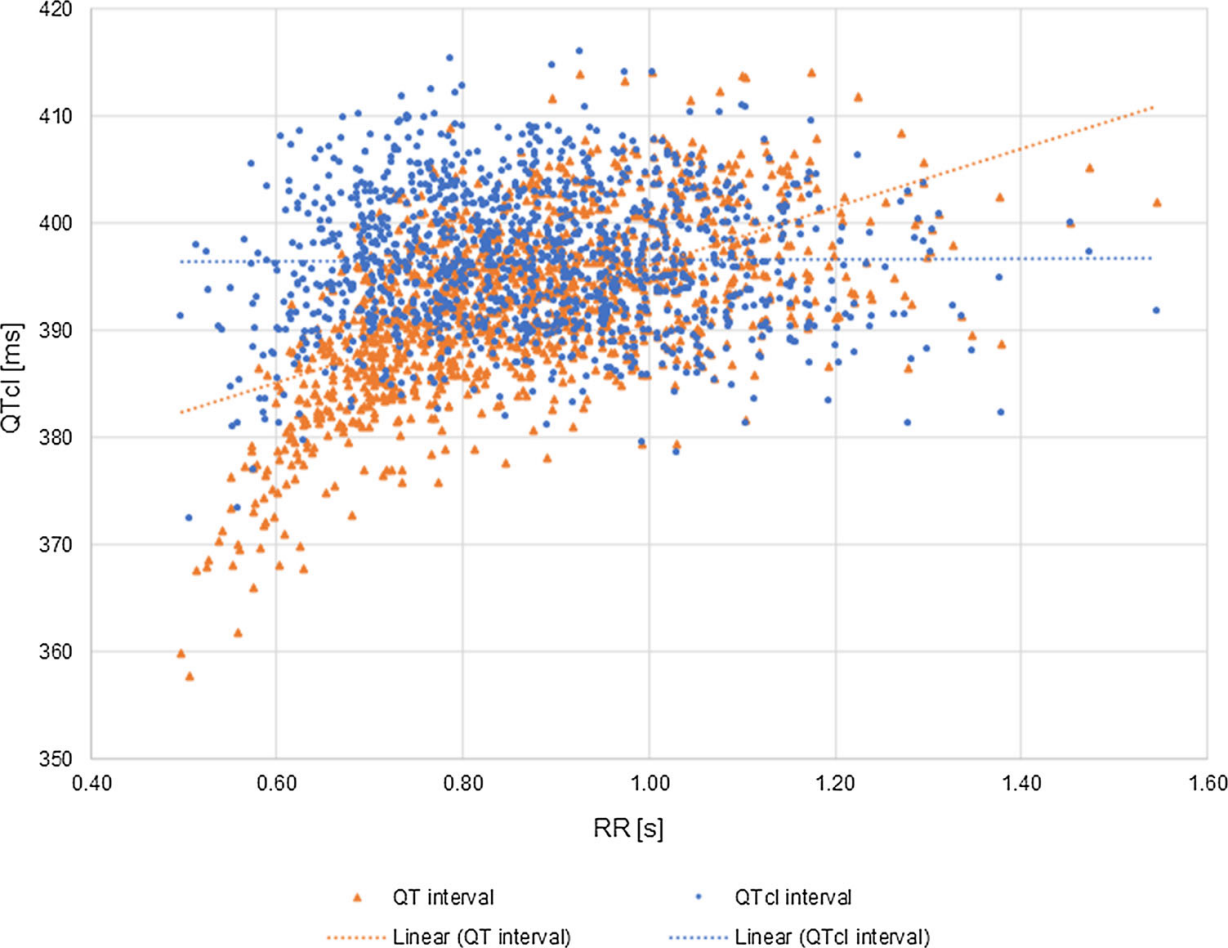
A relationship between QT/QTcI [ms] and RR [s] values extracted from pseudoECG signals simulated for the healthy virtual population. The orange line represents the relationship between native QT values [ms] and the corresponding RR [s] values (orange triangles). Blue line represents the relationship between individually corrected QT values for the heart rate (QTcI [ms]) and the corresponding RR [s] values (blue squares)

**Fig. 4:**
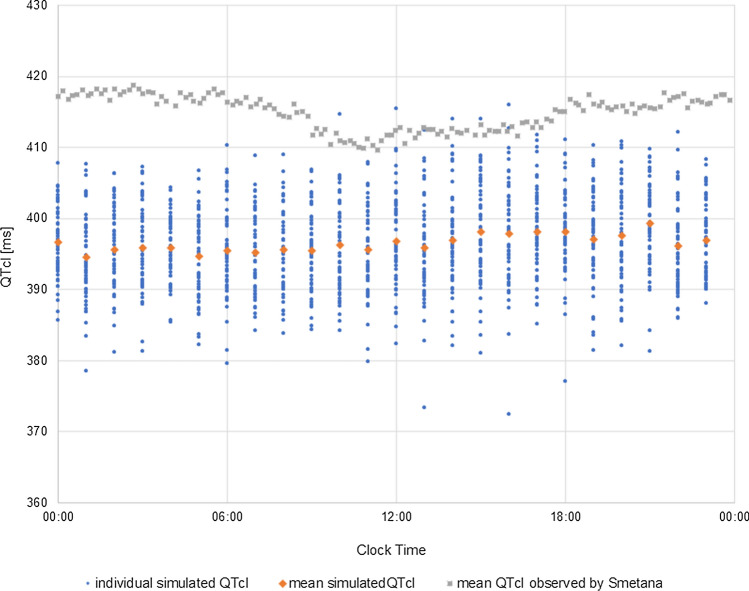
The mean and individual QTcI values [ms] over a 24-h period simulated in CSS and observed by Smetana et al. [12]. Blue dots represent individual simulated QTcI [ms], orange diamonds represent mean simulated QTcI [ms], and grey squares represent mean QTcI [ms] observed by Smetana et al. [12]

Smetana et al. [12] compared a circadian periodicity of QTc as derived by five correction methods, i.e. Fridericia, Bazett, Framingham, Hodges, and individually optimized one. The results differed significantly with different circadian rhythmicity, from non-existent (Bazett) to marked (Fridericia, Framingham, Hodges). In their study, Smetana et al. confirmed the superiority of individually optimized correction formulas over any other universal formulas. As the individual correction limits the influence of actual heart rate on QT interval value, the QT values adjusted this way reflect the real circadian pattern of QTc. Therefore, the individually corrected QT values (QTcI) were calculated (Figs. 3 and 4) as described in the Methods section (Eq. 1).

The relation of QT/QTcI-RR values is presented in Fig. 3. QT/RR scatter diagram illustrates a typical pattern of how QT interval adapts to heart rate changes. The application of the heart correction formula to QT interval length values aims at eliminating this dependency to achieve the correlation between QTc and RR being zero [27]. A scatter plot of QT/QTcI vs. RR in Fig. 3 confirms the independence of these two variables.

he simulated mean and individual QTcI values over a 24-h period are compared with the mean observed QTcI in Smetana study in Fig. 4. The circadian rhythmicity evident though the amplitude of average diurnal changes is small.

**Fig. 5:**
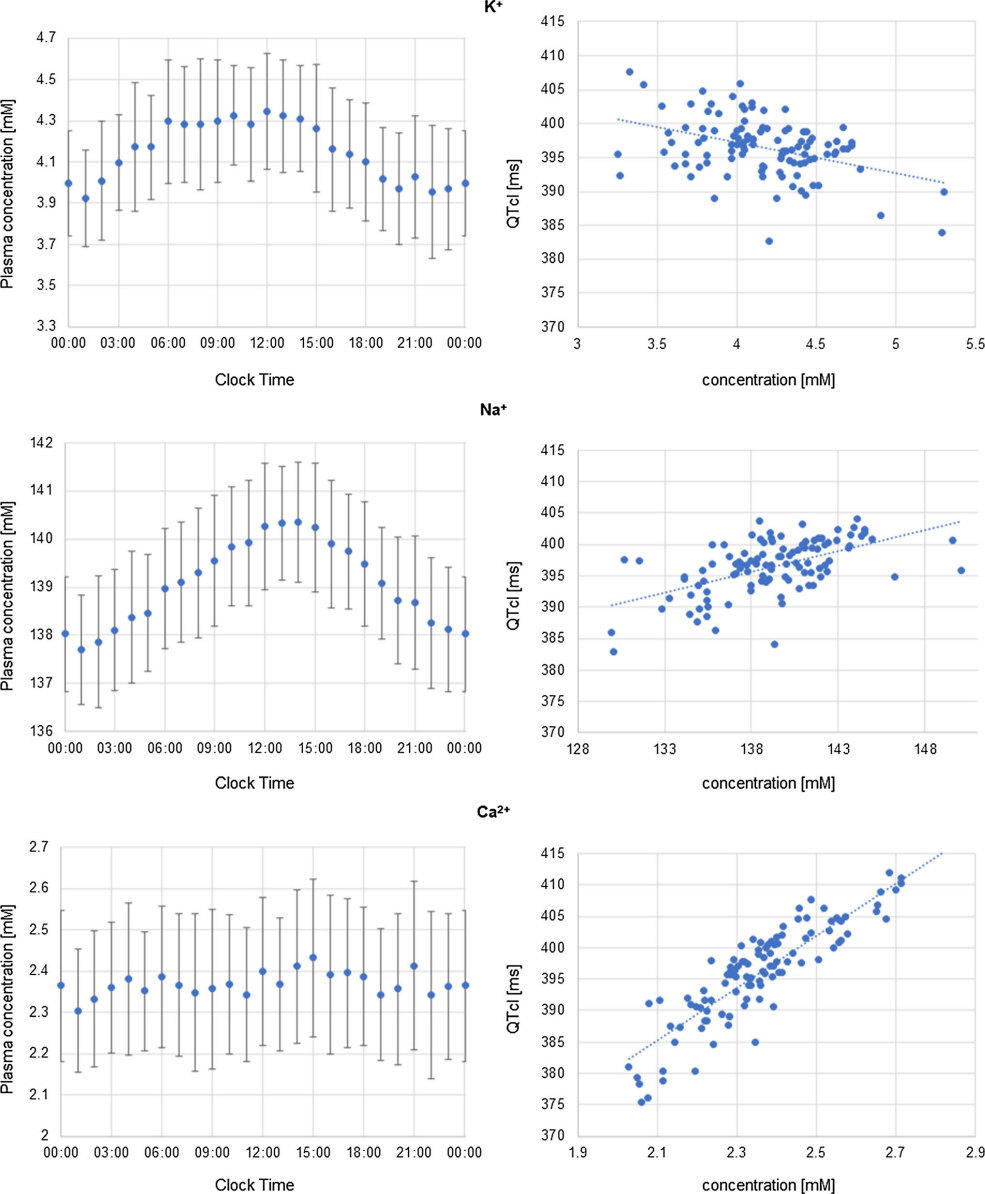
Simulated circadian changes of plasma concentrations of potassium, sodium, or calcium ions and the relationship between ion concentrations and QTcI values. Plots on the left illustrate the diurnal fluctuations of potassium (top panel), sodium (middle panel), and calcium (bottom panel) plasma concentrations [mM] showed as the mean values and standard deviation around a mean. The scatter plots on the right illustrate the relationship between QTcI [ms] values and plasma concentrations of potassium (top panel), sodium (middle panel), and calcium (bottom panel) concentrations [mM]

The individual diurnal variation of QTcI values was reduced as compared to native QT values and ranged from ~ 10 to 33 ms, 17 ms on average. The QT interval duration dependence on RR changes was removed by applying the individual correction model. The remaining deviations resulted from other factors influencing the heart electrical activity and QT interval length, inincluding changes of ion plasma concentrations that show circadian pattern [3, 17] or diurnal variations in cardiac ion channel expression [14, 29]. Based on the analysis of circadian fluctuations of K^+^, Na^+^, and Ca^2+^ ions and the relation of ion concentration with QTcI values it can be postulated that electrolytes balance and its circadian variation is the main factor influencing QT circadian pattern (Fig. 5). Scatter diagrams illustrate correlations between predicted QTcI values and plasma concentrations of potassium, sodium, or calcium ions.

The simulation without the inclusion of the models of circadian HR and ion concentration changes was run for the comparison. When these models were not accounted for, HR and ion concentration values drawn from the predefined distributions to each time point and each subject by the virtual population generator are constant throughout the time of the study. The aim of this experiment was to confirm the ion concentration as the main source of the observed QT variability.

**Fig. 6:**
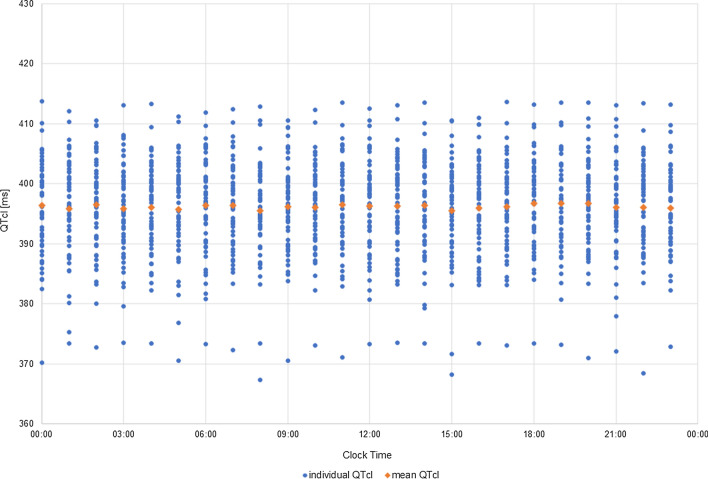
Simulated diurnal changes in QTcI [ms] values in the virtual population when neither the HR circadian changes nor plasma ion concentrations changes were accounted for in the simulation scenario. Blue dots represent individual QTcI values, orange dots represent mean QTcI values

In this case, the simulation results did not reveal any diurnal rhythmicity of either mean or individual QTcI values (Fig. 6). The 24-h period variability of QTcI values for individual subjects was small. On average SD was around 2 ms (range: 0.6–5.6 ms). This result was inconsistent with the general clinical observation that QT interval, even when corrected for circadian changes in the heart rate, displays the diurnal variations [12, 29]. In the previous simulation (Fig. 4), when the models of circadian fluctuations of HR, as well as ion concentrations were taken into account, the individual diurnal variation of QTcI values ranged on average 17 ms which is more expected to be observed in the population.

The comparison of the results of these two numerical experiments has shown the role of inclusion of the models of the circadian variability of the heart rate, potassium, sodium, and calcium concentrations in a simulation of physiological QT length fluctuations. This justifies the utilization of these models in the simulations that are aimed at the assessment of the drug triggered the electrophysiological effect on the QTc interval length.

Tolterodine was chosen as an exemplary drug to assess the importance of incorporation of circadian rhythmicity into the electrophysiological simulations and assessment of drug effects on QT-interval.

**Fig. 7:**
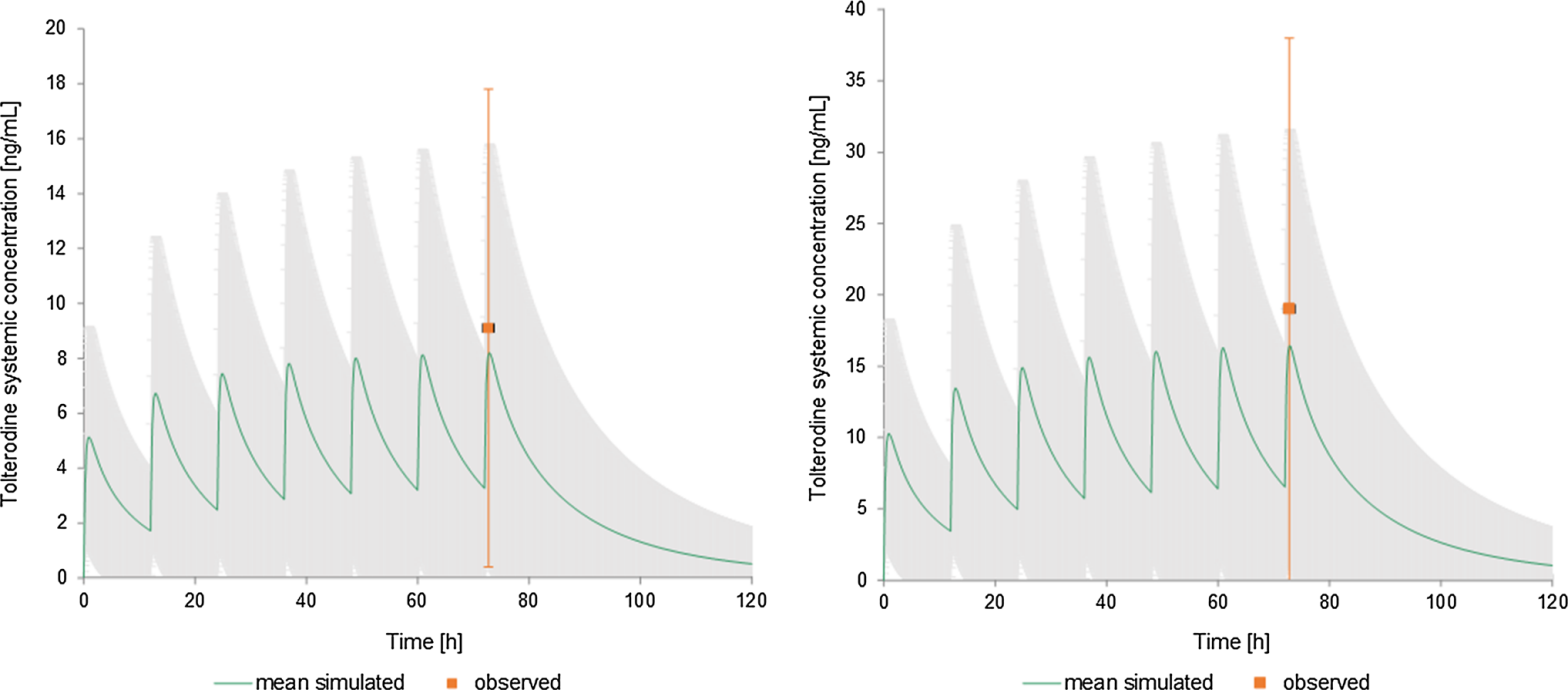
The time-plasma concentration profiles of tolterodine after its administration in a therapeutic dose of 2 mg bid (on the left) or in the supratherapeutic dose of 4 mg bid (on the right). Model-predicted [22] concentrations [ng/mL] are shown as the mean (in green) and 5th and 95th percentile (in grey) and compared with the data observed by Malhotra et al. [21]

The results of tolterodine PK modelling and their discussion were published elsewhere [23]. Figure 7 presents the mean and 5th and 95th percentiles of tolterodine concentrations predicted by the model and their comparison with the data observed by Malhotra [22]. To predict the drug effect on cardiac electrophysiology posed by drug concentration-dependent inhibition of ion currents the simulated individual time-concentration profiles of tolterodine were used. Tolterodine is primarily metabolised by cytochrome P450 2D6 and 3A4. 2D6 isoenzyme exhibits large phenotypical variability in the population due to genetic polymorphism, which in the case of tolterodine pharmacokinetics contributes to the huge inter-subject variability in time-concentration profiles and the maximum concentration in some individuals after therapeutic dose equal to that after supratherapeutic one observed in the population.

Drug effects on four main ion channels were simulated with a simple pore block model as described previously [30]. The electrophysiological changes were simulated accounting for both interindividual and intraindividual variability arising from individual circadian changes of heart rate, potassium, sodium, and calcium ion concentrations. Simulations were run for 10 virtual trials equalling 480 subjects in total.

**Fig. 8:**
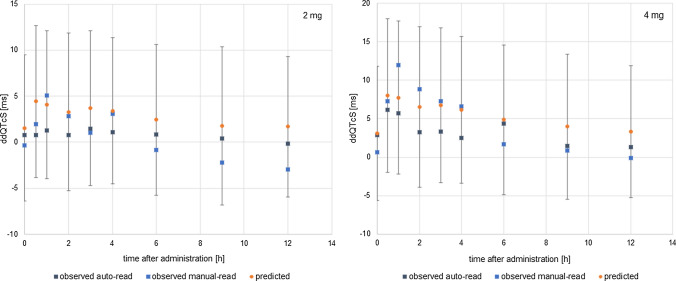
The simulated (orange dots) and the observed, either auto-read (black squares) or manual-read (blues squares) time-matched changes from baseline QTc values (ddQtcS) [ms] on day 4 of tolterodine administration in dose 2 mg bid (on the left) or 4 mg bid (on the right). The data are shown as the mean values, for simulated data with SD

The cardiac safety biomarker i.e. QT interval length and its change was extracted from pseudoECG signal outputted by CSS. To make QT intervals length independent of heart rate the native QTs were corrected with the study-specific formula (QTcS) with the coefficient n of 0.068. In the mimicked study by Malhotra et al. [22] ultimately, the Fridericia correction method was applied to present the results of the study, however other methods, i.e., Bazett and study-specific population correction formula, were also considered. The authors claimed that the exponent in the study-specific correction formula determined for manual-read ECGs was similar to the QTcF exponent implying the comparability of QTcS and QTcF results. Since the Fridericia correction method tends to overcorrect the simulated QT values, the application of the study-specific method in the case of simulation outputs seemed to be justified. The QTc changes were calculated as time-matched differences between QTc during treatment and the baseline, i.e., the situation without a drug. Comparison of simulated results with the clinical observations for the dose of 2 mg and 4 mg, which were either machine- or manual-read, is presented in Fig. 8 and Table 2.

**Table 2:** The simulated and the observed time-matched change from baseline QTc values (study-specific correction) on day 4 of tolterodine administration at the time point corresponding to the maximum concentration of tolterodine (Tmax of 1 h). Data are presented as the mean values, the upper limit of 95% confidence interval (CI_95%_), and standard error of the mean (SE)

	2 mg			4 mg		
	Manual	Auto	Simulation	Manual	Auto	Simulation
Mean	4.5	2	4.1	10.3	8.3	7.7
CI_95%_	9.3	5.8	6.4	15.1	12.2	10.5
SE	2.4	1.9	1.2	2.4	1.9	1.4

*Manual* observed placebo-adjusted change from baseline in manual-read QT/QTc values, *auto* placebo-adjusted change from baseline in machine-read QT/QTc values (details in [22]), *simulation* CSS model predicted values

The clinically observed cardiac effects of tolterodine administered in both, therapeutic and supratherapeutic doses were predicted well by the PK-PD model. In both cases, the value of predicted tolterodine-triggered QTc prolongation achieved in the time point in which tolterodine reached its maximum concentration, lied in between the observed, manual- and machine- read values. The simulation results confirmed that at the recommended dose of tolterodine a 5-ms QTc prolongation could be excluded however, this was not the case when the dose was supratherapeutic.

**Fig. 9:**
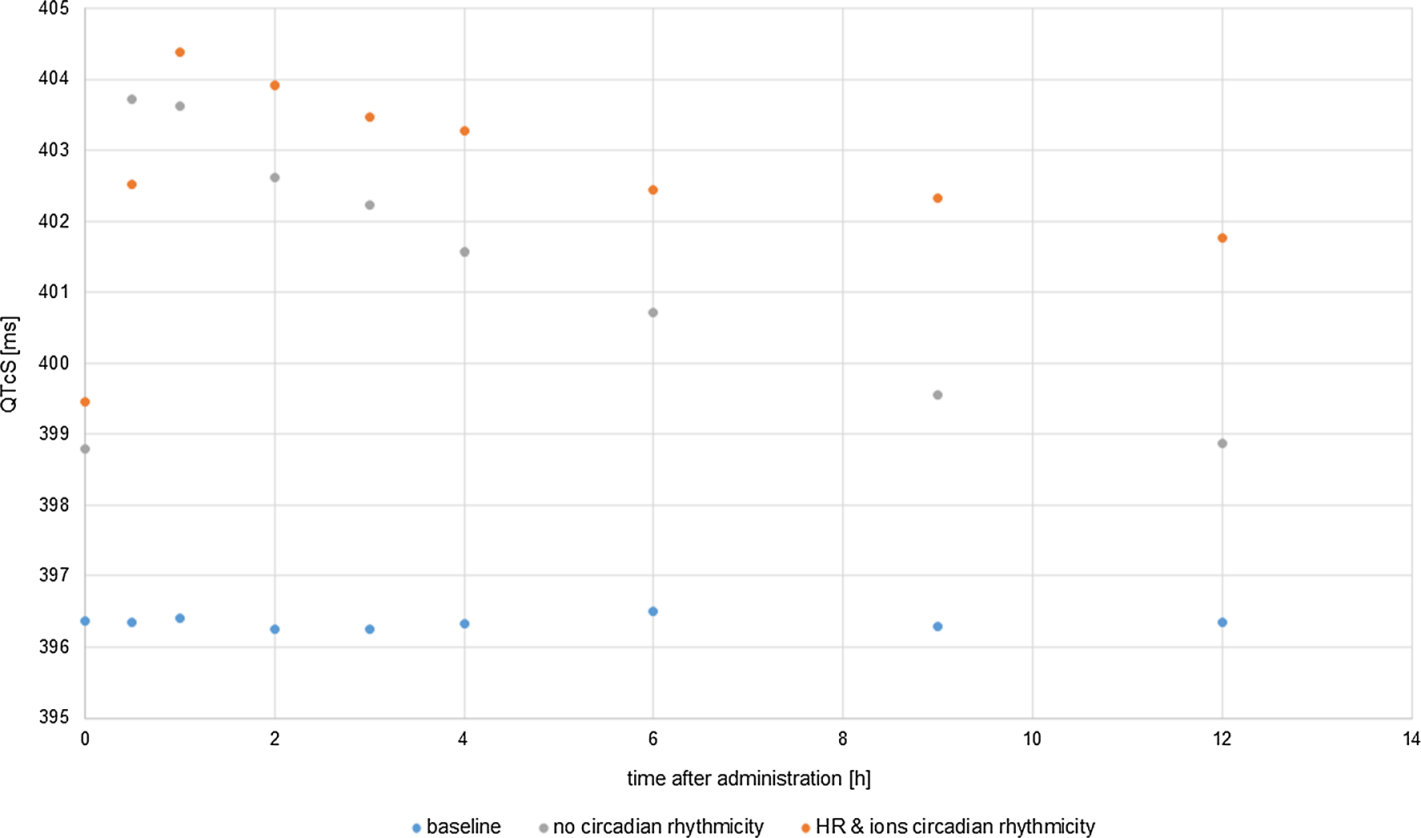
Mean of predicted QTcS values [ms] in time after administration of tolterodine if the HR circadian changes and plasma ion concentrations changes were accounted for in the simulation scenario (orange dots), neither of circadian changes were accounted for in the simulation (grey dots) or in the situation without a drug (blue squares)

To test the influence of the model of circadian ions fluctuation the simulations were rerun assuming that subjects physiological parameters do not change in time (including heart rate and ion concentrations) once generated (Fig. 9).

The mean predicted QTc values, as well as average changes from baseline, did not differ substantially whether the ions changes are accounted for or not. Consequently, circadian ion fluctuations won’t influence the judgement of a compound’s safety. However, for individual subjects their mean QTc values, differed up to 10 ms (range from − 0.7 to 10 ms) and maximal QTc values differ up to 19 ms (range from − 16.2 to 18.6 ms), which would influence the cardiac safety assessment of a given drug to particular individuals. Therefore, when safety is considered at the bed side on individual level, patient specific ion plasma concentrations and their changes may be important.

**Fig. 10:**
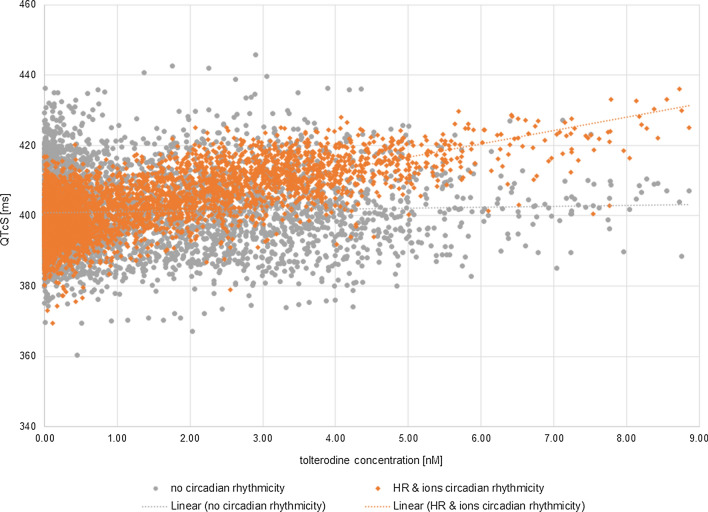
A relationship between simulated individual QTcS [ms] values and tolterodine concentrations [nM]. Orange line represents the relationship between QTcS [ms] and the corresponding tolterodine concentrations [nM] achieved when the HR circadian changes and plasma ion concentrations changes were accounted for in the simulation scenario (orange diamonds). Blue line represents the relationship between QTcS [ms] and the corresponding tolterodine concentrations [nM] achieved when neither of the circadian changes were accounted for in the simulation (grey dots)

Moreover, when the circadian ion variability is not accounted for in the simulation, the predicted QTc changes caused by a drug can be ‘masked’ and overlooked when the drug effect itself is modest, as in the case of tolterodine given in therapeutic dose (Fig. 10). However, in this particular case the observed difference does not have clinical relevance and result of safety assessment as QTc is below the safety threshold regardless of wheather circadian ion variability is considered or not.

## Conclusions

In this two-step study, the performance of the model of circadian HR fluctuations implemented in the CSS software was evaluated and the importance of incorporation of circadian rhythmicity into modelling and simulation of the drug cardiac safety was assessed. The results of the simulations were in line with the clinical observations showing the sinusoidal pattern of the diurnal RR and QT changes. Since physiologically QT interval adapts to RR fluctuations, the analysis of the simulation results led to the hypothesis that the electrolytes balance and its circadian variability is the main factor influencing QT circadian pattern. The numerical experiment on simulation of the cardiac effect of two doses of tolterodine confirmed that CSS can be useful in the assessment of drug-triggered cardiac effects which depend on the drug concentration and the time of a day as well. The diurnal ion changes when taken into account increased an intra-subject variability of predicted QTc prolongation effect which is in line with the clinical observation.. To sum up, the study results have shown influence of the models of the circadian variability of the heart rate, potassium, sodium, and calcium concentrations implemented in the CSS software on QT prediction and have justified the use of these models in the simulations that are aimed at the assessment of the drug triggered electrophysiological effect on the QTc interval length. However, when time-matched baseline correction was used to calculate drug-induced QT change, the circadian fluctuations of ions did not have a decisive effect on the conclusion upon drug effect.
